# Interpreting Deep Neural Networks in Diabetic Retinopathy Grading: A Comparison with Human Decision Criteria

**DOI:** 10.3390/life15091473

**Published:** 2025-09-19

**Authors:** Sangeeta Biswas, Md. Ahanaf Arif Khan, Md. Hasnain Ali, Johan Rohdin, Subrata Pramanik, Md. Iqbal Aziz Khan, Sanjoy Kumar Chakravarty, Bimal Kumar Pramanik

**Affiliations:** 1 Faculty of Engineering, University of Rajshahi, Rajshahi 6205, Bangladeshsprmnk@ru.ac.bd (S.P.); iqbal_aziz_khan@ru.ac.bd (M.I.A.K.); sanjoy.cse@ru.ac.bd (S.K.C.); bkp@ru.ac.bd (B.K.P.); 2 Faculty of Information Technology, Brno University of Technology, 61200 Brno, Czech Republic; rohdin@fit.vutbr.cz

**Keywords:** deep neural network, d iabetic retinopathy classification, Grad-CAM, fundus image, integrated gradients

## Abstract

Diabetic retinopathy (DR) causes visual impairment and blindness in millions of diabetic patients globally. Fundus image-based Automatic Diabetic Retinopathy Classifiers (ADRCs) can ensure regular retina checkups for many diabetic patients and reduce the burden on the limited number of retina experts by referring only those patients who require their attention. Over the last decade, numerous deep neural network-based algorithms have been proposed for ADRCs to distinguish the severity levels of DR. However, it has not been investigated whether DNN-based ADRCs consider the same criteria as human retina professionals (HRPs), i.e., whether they follow the same grading scale when making decisions about the severity level of DR, which may put the reliability of ADRCs into question. In this study, we investigated this issue by experimenting on publicly available datasets using MobileNet-based ADRCs and analyzing the output of the ADRCs using two eXplainable artificial intelligence (XAI) techniques named Gradient-weighted Class Activation Map (Grad-CAM) and Integrated Gradients (IG).

## 1. Introduction

Diabetic Retinopathy (DR) is a microvascular complication of diabetes. In the retinal blood vessels of diabetic patients, high glucose levels damage the pericyte cells that wrap around the capillaries in the retina, as well as the endothelial cells that regulate exchanges between the bloodstream and the surrounding tissues. The destruction of essential cells causes retinal blood vessels to lose the ability of maintaining normal vascular tension, grow unstable, and be easily damaged by oxides, resulting in insufficient blood circulation, vascular leakage, retinal hemorrhages, and ultimately leading to DR [1,2,3]. It is one of the most serious and frequently occurring complications in diabetic patients. According to the International Diabetes Federation (IDF), approximately 103.12 million out of 537 million diabetic patients had DR in 2021 [4,5]. It is a leading cause of partial or full vision loss globally [5,6,7]. Besides vision impairments, there is evidence that DR is associated with other micro- and macrovascular complications of diabetes such as subclinical atherosclerosis [8,9,10,11], cardiovascular disease [12,13,14,15,16], cerebrovascular incident [17,18,19,20,21], cognitive impairment [22], dementia [23], and high risk of mortality [19,24,25].

DR is not curable; however, early treatment can prevent, delay, or reduce vision loss. Regular retina checkups can ensure that DR patients receive treatment at appropriate times. With a global rise in diabetic patients and the shortage of Human Retina Professionals (HRPs), many patients do not receive a proper diagnosis on a regular basis in most countries, especially in third-world countries. Capturing fundus images requires the physical presence of the patients at the eye centers. In third-world countries, the number of eye centers is inadequate and often unreachable for many patients. Moreover, manually detecting DR by examining fundus photographs is time-consuming. These obstacles reduce the possibility of regular checkups for many diabetic patients. Therefore, many diabetic patients remain at risk of vision loss without accessible and affordable solutions, which inspires researchers to develop digitized fundus image-based Automatic Diabetic Retinopathy Classifiers (ADRCs). By providing automated imaging, DR grading, and reporting within minutes during a diabetic patient’s regular exam with minimal involvement of short-term trained operators, and by recommending patients having referable DR (such as moderate DR, severe DR, and proliferative DR) for further detailed examinations, ADRCs reduce the burden of retina specialists.

At the beginning, ADRCs were mainly based on basic image processing techniques [26,27]. Then, non-neural network-based machine learning techniques were proposed as ADRCs [28,29,30,31], and gradually neural network-based ADRCs drew the main attention. Even though neural network-based ADRC was proposed in 1996 [32], it gained popularity after 2016. Since then, Deep Neural Network (DNN)-based algorithms [33,34,35,36,37,38,39,40,41,42,43,44,45,46,47,48,49,50,51,52,53,54,55] have been dominating ADRC development.

A clear understanding of the criteria underlying DR grading is crucial for an HRP, such as a retina doctor, consultant, or specialist, to ensure reliable use of ADRCs in automatic DR grading and to prevent incorrect treatments. This is why the interpretability of DNN-based systems is an important issue. If it turns out that humans and machines use different criteria, they probably could complement each other, or in the longer perspective, machines can be improved by learning to use the same criteria humans use. If they already use the same criteria as humans, then the analysis of their domain robustness could be reduced to analyzing the domain robustness of the segmentation of individual lesions. Even though so many DNN-based algorithms are published for DR classification, very few works ([37,43,51]) have shown which parts of fundus images contribute to the final decision on DR grades. Moreover, none of these works determined whether the decision-making criteria of ADRCs are similar to those of HRPs. In this work, we investigate whether a DNN-based ADRC that classifies DR into different grades considers the same criteria as HRPs. We believe the findings from our investigation will help developers improve the accuracy of ADRCs while assisting HRPs to better understand how ADRCs operate.

We describe currently applicable DR severity classification schemes in Section 2 and previous works on interpretability issues in Section 3. In Section 4, we outline our approach for analyzing ADRC’s decision. Our experimental setup is described in Section 5. In Section 6, we present our ADRCs’ performance. In Section 7, we analyze our ADRCs’ decisions, and finally in Section 8, we draw our conclusion.

## 2. DR Severity Classification Schemes for HRPs

In 1890, Hirschberg proposed the earliest known classification for DR [56], which then evolved as human understanding of disease pathophysiology was improved, methods of imaging were changed, methods of DR assessment were updated, and effective treatments were developed. After going through many modifications ([57,58,59,60,61,62,63]) for more than 130 years, the severity classification of DR has come to a stage where DR is mainly divided into two grades considering the absence or presence of new, tiny, and abnormal blood vessels: (1) Non-Proliferative DR (NPDR) and (2) Proliferative DR (PDR). Various signs such as microaneurysms, hard and/or soft exudates, venous caliber abnormalities, venous sheathing, perivenous exudate, arteriolar abnormalities, intra-retinal microvascular abnormalities (IRMAs), and arteriovenous nicking are included in NPDR. Conversely, retinal or disc neovascularization, fibrous proliferation, retinal detachment, and preretinal and vitreous haemorrhage are included in PDR.

By subdividing NPDR in different ways, different DR grading schemes were proposed, such as Early Treatment of Diabetic Retinopathy Study (ETDRS) [63], International Clinical Diabetic Retinopathy (ICDR) [64], Scottish scheme [65], and National Health Service (NHS) England scheme [66], to accurately describe the progression of DR, quantify its severity, and predict the risk of progression. Nowadays, the ETDRS severity scale, proposed in 1991, is primarily utilized in research and intervention studies worldwide. However, it is not suitable for routine clinical use. The NHS and Scottish grading schemes have limited use outside England, Wales, and Scotland.

In 2002, the ICDR grading scheme was proposed by simplifying the ETDRS severity scale. Due to its convenience and ease of adoption, the ICDR grading scheme is by far the most common DR grading system in clinical use worldwide. The ICDR grading scheme defines the DR severity levels as follows: DR-0 (No DR) indicates no evidence of retinopathy or related abnormalities; the presence of only microaneurysms characterizes DR-1 (Mild DR); DR-2 (Moderate DR) includes one or more microaneurysms, retinal dot or blot hemorrhages, hard or soft exudates, without signs of severe retinopathy; DR-3 (Severe DR) requires more than 20 intraretinal hemorrhages in each of the four quadrants, definite venous beading in at least two quadrants, or prominent intraretinal microvascular abnormalities in at least one quadrant, but no signs of proliferative retinopathy; finally, DR-4 (PDR) is defined by the presence of neovascularization, or vitreous or preretinal hemorrhages. Many retina datasets based on the ICDR scale are publicly available for researching automatic DR classification, such as the Indian Diabetic Retinopathy Image Dataset (IDRiD), Kaggle EyePACS Diabetic Retinopathy Detection dataset (Kaggle EyePACS), and Messidor-2.

## 3. Previous Works on Interpretability of ADRCs

The accuracy of predictions, as well as the interpretability of the reasons behind them, is essential for ADRCs. Self-explanatory ADRCs enable HRPs to compare the information reported by an ADRC with their own knowledge, increasing the probability of an accurate diagnosis, which may significantly influence a patient’s treatment. Even though many DNN-based ADRCs have been proposed since 2016, very few studies (e.g., [35,36,37,40,43,49,51,53]) have addressed the interpretability issue of DNN-based ADRCs. All these works generated heatmaps using eXplainable Artificial Intelligence (XAI) techniques and highlighted regions important for final decision-making by overlaying heatmaps on fundus images.

Gargeya et al. [35] generated heatmaps with the help of the Classification Activation Maps (CAMs) [67]. To generate heatmaps, they implanted a convolutional visualization layer at the end of their network. By overlaying the heatmap on the fundus image, the authors demonstrated that the features of PDR, such as retinal hemorrhage, hard exudates, and neovascularization, can be highlighted with the help of the visualization layer of their ADRC. Even though they provided evidence of their method’s ability to highlight severely affected pathologic regions, they did not provide any evidence that their method is capable of highlighting regions affected by mild or moderate DR. Even in their example image (Figure 5 in [35]), many DR-affected regions are not highlighted.

Quellec et al. [36,49] modified CAM to produce heatmaps. Wang et al. [37] proposed Regression Activation Maps (RAMs), an updated version of CAM, to generate heatmaps. They provided evidence that their proposed RAM learned to highlight DR severity-specific signs, such as the narrowing of the retinal arteries for mild-conditioned DR patients and balloon-like structures for severe DR. Gao et al. [40] also utilized CAM to generate heatmaps. They provided evidence that their model focused on primary lesions during classification by providing heatmaps for fundus images affected by severe DR and PDR (Figure 6 in [40]). However, they did not provide any evidence for mild DR or moderate DR. Therefore, it is unclear where their ADRCs focused during decision-making.

Sayres et al. [43] applied Integrated Gradients (IG) [68] to generate heatmaps for assisting DR graders. They visualized the highlights over the fundus image by converting it to grayscale and overlaying the explanation heatmap as a semi-transparent green heatmap. They investigated the accuracy, speed, and confidence of HRPs if they are provided model-predicted DR scores and heatmaps. They observed that grades plus heatmaps improved accuracy for patients with DR but reduced accuracy for patients without DR.

Torre et al. [51] developed score maps to quantify pixel contributions to the final classifications. They followed an approach similar to pixel-wise decomposition [69,70]. Their study concluded that the input space and the receptive fields of each layer were the two main factors that contributed to the output score. They generated score maps by propagating back the score part that depends on the previous input in each layer. Their investigation provided evidence that the combination of micro-information from input space maps with macro-information obtained from intermediate scores was the key to understanding the results and helping medical personnel improve their diagnosis processes.

Al-Antary et al. [53] proposed to use an attention map, one kind of heatmap, to demonstrate the importance of the input area for DR classification. Unlike in [35,36,37,40,43,49,51], the authors utilized attention maps during the training of their ADRC to enhance its focus on informative image regions, specifically highlighting the diseased parts in the fundus image while placing less emphasis on the normal regions. They produced attention maps from multi-level, multi-scale pyramid representations of fundus images. They demonstrated the importance of the input area for recognizing DR by providing two attention maps extracted for normal and moderate DR in Figure 11 in [53]. However, they did not provide evidence to indicate whether the highlighted area in the attention map always contains DR-grade specific lesions or biomarkers.

## 4. Proposed ADRC Decision Analysis

The previous works mentioned in Section 3 used XAI techniques to aid HRPs, whereas our work use them to analyze whether DNNs use the same information, i.e., the criteria specified in the ICDR grading scheme, as HRPs for DR classification. For our analysis, we followed three stages: building ADRCs, building segmentation models, and analyzing ADRCs’ decisions.

Stage A. Building ADRCs
  1. First, we prepared training, validation, and test sets by splitting three DR severity classification datasets: Kaggle EyePACS [71,72], IDRiD-DR [73], and Messidor-2 [74].
  2. We trained DNN-based ADRCs for classifying five severity levels of DR.
  3. Then, we analyzed the DR classification ability of our ADRCs by evaluating them on the test sets using different evaluation metrics and confusion matrices.
Stage B. Building Segmentation Models
  1. We prepared training, validation, and test sets using three datasets: IDRiD [73], E-ophtha [75], and PALM [76].
  2. We trained six binary segmentation models to segment the optic disc, macula, and four types of abnormalities, including microaneurysms, hard exudates, soft exudates, and hemorrhages. We used these segmentation models to generate segmentation masks for each retina image.
Stage C. Analyzing ADRCs’ Decisions
  1. To analyze the interpretability of our ADRC, we first generated heatmaps for all fundus images of the test set of the IDRiD-DR dataset using Gradient-weighted Class Activation Map (Grad-CAM) and Integrated Gradients (IG).
  2. We overlayed the heatmap on the fundus images and manually identified and recorded where DNN-based ADRCs emphasize while making decisions about the DR severity levels, i.e., DR classes.
  3. We automatically determined, with the aid of segmented masks and heatmaps, which parts of the fundus images played a crucial role in the final DR-level detection. For this, we first extracted the areas where XAI technique-generated heatmaps had the highest activation. Next, we performed a pixel-wise logical “AND” operation between these high-activation regions, obtained by applying percentile-based thresholding to the heatmaps and the corresponding binary masks generated by our segmentation models. After that, we determined the overlapped area between the model’s predicted regions of interest and the actual pathological areas. Formally, for each image *i*, we define the following:
    - $H_{i}$: the XAI heatmap (Grad-CAM or IG) for image *i*, normalized to the range [0,1].
    - $M_{i}$: the binary segmentation mask for image *i*, where 1 indicates a lesion region.
    - $T^{\mathrm{GC}}$, $T^{\mathrm{IG}}$: the thresholds corresponding to the 90th and 97th percentiles of $H_{i}$, for Grad-CAM and IG, respectively.
    The binary activation map $A_{i}$ is defined as follows:
    $$A_{i}\left(x,y\right)=\left\{\begin{array}{cc} 1, & \mathrm{if}\;H_{i}\left(x,y\right)\geq \;T\\ 0, & \mathrm{otherwise} \end{array}\right.$$
    where $T=T^{\mathrm{GC}}$ or $T^{\mathrm{IG}}$, depending on the method.
    The intersection mask is computed as follows:
    $$I_{i}=A_{i}\wedge M_{i}$$
    If the intersection contains any non-zero values (i.e., $\sum_{x,y}I_{i}\left(x,y\right)>0$), it implies that the heatmap has activated a pathological region. We count the number of such instances across all images and analyze the results.
  4. We also applied a semi-automatic approach where we generated Grad-CAM and IG heatmaps from the Retinal-Lesions dataset and performed the same mentioned operation between the highest activation regions and their provided segmentation masks for eight types of lesions: microaneurysm, cotton wool spots, hard exudate, retinal hemorrhage, preretinal hemorrhage, vitreous hemorrhage, fibrous proliferation, and neovascularization.

## 5. Experimental Setup

### 5.1. Hardware and Software

We used a computer system equipped with an AMD Ryzen 5 3500X CPU, 32 GB of DDR4 memory, and a single NVIDIA RTX 3090 GPU with 24 GB of GDDR6 VRAM for conducting our experiments. Our system ran Ubuntu 22.04 LTS with Python 3.11. We utilized TensorFlow v2.15, along with the Keras API and other Python-based libraries, including OpenCV, Scikit-Learn, and Matplotlib.

### 5.2. Datasets

We used six datasets in total, as listed in Table 1. We only utilized free and publicly available datasets in our work. No additional data collected from hospitals, clinics, or other external sources were used.

#### 5.2.1. Short Summary of Datasets

The Kaggle EyePACS Diabetic Retinopathy Detection (Kaggle EyePACS in short) dataset is one of the most popular datasets for detecting DR [71]. It was made publicly available on the Kaggle community platform for a competition on DR detection sponsored by the California Healthcare Foundation. Fundus images of this dataset were provided by EyePACS [72]. The images in this dataset are high-resolution color fundus photographs captured using different models and types of specialized imaging equipment with diverse resolutions, ranging from 211×320 to 3456×5184 pixels.

The Indian Diabetic Retinopathy Image Dataset (IDRiD) [73] contains images from clinical examinations conducted at an eye clinic in India. Images of this dataset are split into three subsets: (1) Disease Grading, (2) Localization, and (3) Segmentation. The subset of the IDRiD dataset intended for ‘Disease Grading’ contains a total of 516 high-quality fundus images for DR classification. Each image was captured with a 50° field of view and a resolution of 4288×2848 pixels, stored in JPEG format. We denote this subset as IDRiD-DR.

Messidor-2 contains a total of 1748 macula-centered fundus images collected from 874 individuals diagnosed with diabetes [74]. The images have a 45° field of view, and the dataset comes with a spreadsheet containing image pairs but does not include annotations for DR severity classification. However, third-party annotations are available.

The ‘Segmentation’ subset of the IDRiD dataset consists of 81 high-resolution macula-centered fundus images with a resolution of 4288×2848 pixels and a $45^{\circ }$ Field of View (FOV). The images were captured using a Kowa VX-10 Alpha digital fundus camera. The images are divided into two distinct sets for training and testing, containing 54 and 27 images, respectively. The dataset provides pixel-wise annotations for four types of lesions: hard exudates, soft exudates, hemorrhages, and microaneurysms, as well as the optic disc. We denote this subset as IDRiD.

E-ophtha [75] is a publicly available dataset of fundus images designed for the segmentation and detection of DR lesions. It consists of two subsets: e-ophtha EX for hard exudates and e-ophtha MA for microaneurysms. The dataset contains a total of 463 images, among which 47 and 148 images contain annotations for hard exudates and microaneurysms, respectively. The images have varying resolutions and FOVs and are widely used for evaluating segmentation models.

The PAthoLogic Myopia (PALM) [76] dataset is designed for the segmentation and detection of pathological myopia (PM) and associated lesions in fundus images. It contains 400 images with a resolution of 1444×1444 pixels, captured with a $45^{\circ }$ FOV. The dataset includes pixel-wise annotations for the optic disc and three types of lesions: retinal detachment, atrophy, and myopic maculopathy. The images are divided into a training set of 320 images and a test set of 80 images.

The Retinal-Lesions [77] dataset includes 12,252 fundus images sourced from the Kaggle EyePACS dataset and some local hospitals. The images were annotated for eight lesion types, including microaneurysms, intraretinal hemorrhages, hard exudates, cotton-wool spots, vitreous hemorrhages, preretinal hemorrhages, neovascularization, and fibrous proliferation. To ensure high-quality labeling, 45 ophthalmologists participated in the annotation process. The images were standardized by cropping to FOV and resizing to a 896×896 pixel resolution. A subset of this dataset, comprising a total of 1593 images and 4143 masks, is available upon request. Among 1593 images, 953 have the same grades as in the Kaggle EyePACS dataset, while 640 images have different grades. The images were from both the training and testing sets of the Kaggle EyePACS dataset. In addition to the lesion masks, adjusted labels are also provided for each image.

#### 5.2.2. Datasets for Building ADRCs

For training and testing ADRCs, we used three publicly available datasets: Kaggle EyePACS [71,72], IDRiD-DR [73], and Messidor-2 [74]. Each dataset contains images categorized into five distinct retinopathy grades, according to the ICDR grading scheme [64]. Table 2 shows each dataset’s number of images per DR grade. We used the first two datasets for both training and testing because they have distinct training and test sets. The third dataset (Messidor-2) lacks a separate training set and test set. Therefore, we used it solely for testing, as in previous works on DR classification [33,35,41,45]. For the Messidor-2 labels, we utilized the annotations provided by Krause et al. [38]. These annotations are accessible on the Kaggle platform.

#### 5.2.3. Datasets for Building Segmentation Models

For training segmentation models, we used three datasets: IDRiD [73], E-ophtha [75], and PALM [76]. We merged and shuffled the IDRiD (both the training set and the test set), E-ophtha, and PALM datasets. Of the total images, 68% were used for training, 15% for validation, and 17% for testing. The distribution of images is shown in Table 3.

#### 5.2.4. Datasets for Analyzing ADRCs’ Decision

For analyzing the decisions made by our trained ADRCs, we used the IDRiD-DR test set and the Retinal-Lesions [77] dataset. We split the 1593 images of the Retinal-Lesion dataset into two subsets: Retinal-Lesion (A) and Retinal-Lesion (B), based on the Kaggle EyePACS subsets from which they were originally derived. There were 977 images in the Retinal-Lesion (A) subset and 616 images in the Retinal-Lesion (B) subset. As shown in Table 4, the Retinal-Lesion (A) set contains images of all DR grades. On the contrary, the Retinal-Lesion (B) set does not include images of DR-0 and DR-1 grades. We used the original grades provided in the Kaggle EyePACS dataset for our analysis. For manually analyzing the ADRCs’ decisions, we only utilized the IDRiD-DR test set, as it is sufficiently small to allow for manual analysis. For automatic analysis, we used the IDRiD-DR test set and both of the Retinal-Lesion subsets.

### 5.3. Training Setup for ADRCs

**Figure 1:**
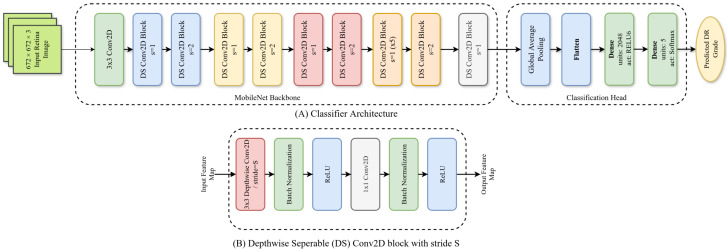
Architecture of our ADRCs. Here, (**A**): overall architecture; (**B**): architecture of a single Depthwise Separable (DS) block.

We used a MobileNet [78] as the backbone of our ADRCs. It was pretrained on the ImageNet-1k dataset for classifying 1000 types of objects. We discarded the fully connected layers of the pretrained MobileNet. We added two fully connected layers: a hidden layer with 2048 units having ReLU6 activation and a classification layer with softmax activation (as shown in Figure 1).

We first resized the fundus images to a 672×672 resolution and then normalized the pixel values to the range of −1 to +1. Initially, the MobileNet backbone of our model was frozen, and only the weights of the newly added layers were adjusted for 50 epochs. Then, the backbone was unfrozen, enabling the entire model’s weights to be updated. Then, the model was trained for another 200 epochs. Its performance was monitored using the accuracy of the validation set. The validation set consisted of 10 and 5 images per class for the Kaggle EyePACS and IDRiD-DR datasets. We used early stopping with a patience of 50 epochs and a batch size of 16. We employed the Adam optimizer with an initial learning rate of 0.0001 to minimize the cross-entropy between the labels and targets. We also used a Plateau learning rate scheduler with a patience of 20 epochs and a reduction factor of 0.8. The weights of the best-performing epoch were saved and later used for the final evaluation of the model. We also employed data augmentation techniques, such as random flipping, rotation, and adjustments in brightness, contrast, and saturation, to improve the generalization of our models.

### 5.4. Training Setup for Segmentation Models

**Figure 2:**
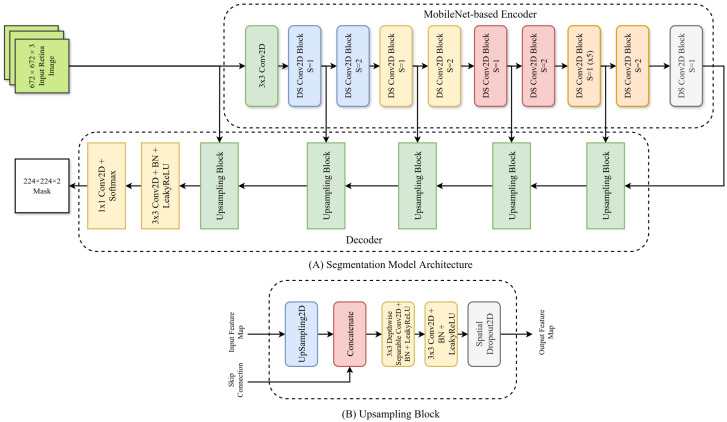
Architecture of our U-net-based segmentation model with the MobileNet encoder and a customized decoder. Here, (**A**): overall segmentation model architecture; (**B**): a single upsampling block.

We trained a total of six segmentation models: four for segmenting lesions (MA, EX, HE, SE) and two for segmenting the optic disc and the macula. Our segmentation models were based on the U-Net [79] architecture. It had a MobileNet [78] encoder and a customized decoder (see Figure 2A). The MobileNet encoder was pretrained on the ImageNet-1k dataset. The customized decoder had five Upsampling blocks (Figure 2B). Each block contained an UpSampling2D layer, a concatenation layer, a Depthwise Separable Conv2D block (3×3 Depthwise Convolution, Batch Normalization, LeakyReLU), a Conv2D block (3×3 Convolution, Batch Normalization, LeakyReLU), and a Spatial Dropout2D layer with a rate of 10%. The output of the last Upsampling blocks was passed to another Conv2D block, and the output layer was a 1×1 pointwise convolution layer with softmax activation.

The fundus images were preprocessed by applying Contrast Limited Adaptive Histogram Equalization (CLAHE) with a clip limit of 5 and tile grid size of 12×12. The images’ pixel values were then normalized to a range of −1 to +1. For predicting lesion masks, the preprocessed images and lesion masks were resized to 1120×1120 resolution, and a total of 25 pairs (224×224 resolution each) of non-overlapping patches were extracted from each image-mask pair. For segmenting the optic disc and the macula, whole images resized to 224×224 resolution were used as a single patch. The patches were shuffled and grouped into batches, each containing 32 samples. Data augmentation techniques, random vertical and horizontal flips, rotations, translations, scaling, perspective changes, and adjustments to brightness and contrast were used to improve generalization.

We trained segmentation models in two stages. Initially, we trained by updating only the decoder weights, keeping the pretrained encoder weights frozen for 50 epochs. Then, the entire model was trained for an additional 250 epochs. In both stages, we used the cross-entropy loss function with a label smoothing value of 0.1. We used the AdamW optimizer with initial learning rates and weight decays of $1\times 10^{-3}$ and $4\times 10^{-3}$ in the first stage, and $5\times 10^{-4}$ and $1\times 10^{-4}$ in the second stage. We used a plateau learning rate scheduler with a patience of 15 epochs and a factor of 0.8.

Our goal was not to benchmark the segmentation models against previous works, but rather to develop workable models for subsequent interpretability analysis. To this end, we trained the segmentation models twice. In the first round, only the training set was used for model training, while the test set was used to evaluate the generalization ability of our models. After confirming that the models achieved reasonable performance, we retrained them on a combined set of the training and testing data. In both cases, the same validation set was used for tuning the hyperparameters.

### 5.5. Evaluation Metrics

For our ADRCs, we used accuracy, F1 score, specificity, and Area Under the Receiver Operating Characteristic Curve (AUC-ROC) as evaluation metrics. For our segmentation models, we used Mean Intersection Over Union (MIoU) as an evaluation metric. We also used Cohen’s kappa coefficient to assess the agreement between our manual and automatic analyses. Accuracy is a measure of the overall correctness of a model. It is calculated as the proportion of correctly classified images over the total number of images. The F1 score metric is the harmonic mean of precision and recall. It provides a balance between precision and recall and is especially useful when the class distribution is imbalanced. Specificity is the proportion of actual negatives that are correctly identified as negatives. It is essential for assessing the model’s performance when false positives are costly. The AUC-ROC curve plots the true positive rate versus the false positive rate for various threshold values. The AUC-ROC value ranges from 0 to 1, with higher values indicating better discriminatory performance of the model. IoU, also known as the Jaccard index in multi-label classification, compares a set of inference labels of a sample to the corresponding set of ground-truth labels. The Cohen’s kappa coefficient is a statistical measure that quantifies the level of agreement between two raters or classification methods, while correcting for the agreement that could occur purely by chance. It ranges from −1 to +1, where values closer to 1 indicate strong agreement, values near 0 indicate agreement by chance, and negative values reflect systematic disagreement.

### 5.6. Interpretation Using Explainable AI

We used two XAI techniques named Gradient-weighted Class Activation Map (Grad-CAM) proposed by Selvaraju et al. in [80] and Integrated Gradients (IG) proposed by Sundararajan et al. in [68] to visualize the parts of an input fundus image that influence the decision of an ADRC. For Grad-CAM, we computed the gradient of the predicted DR class for an input fundus image with respect to the activation of the last convolutional layer (before the global average pooling layer) of our ADRC. For IG, we computed the average gradients of the model’s output with respect to each input pixel along a straight-line path from a baseline image to the actual fundus image. The final attribution score for a pixel is obtained by multiplying this average gradient by the difference between the input pixel and its baseline value.

## 6. Results and Discussion

### 6.1. Results of DR Severity Classification

The performance of our ADRCs, trained on two datasets (Kaggle EyePACS and IDRiD-DR) and evaluated across three test datasets (Kaggle EyePACS, IDRiD-DR, and Messidor-2), is summarized in Table 5. In general, the results show that the ADRCs performed best when the training and test sets came from the same dataset. Conversely, when an ADRC was tested on other datasets, we observed a decline in performance. This may indicate some inter-dataset variability, i.e., there are noticeable differences in the images across different datasets. Some reasons that may be behind these differences are the use of different fundus cameras, different setups of image acquisition systems and environment (e.g., varying lighting conditions), involvement of different camera operators, different ethnicities of subjects from which images were captured, different aged subjects, and even the differences in image storing methods.

**Figure 3:**
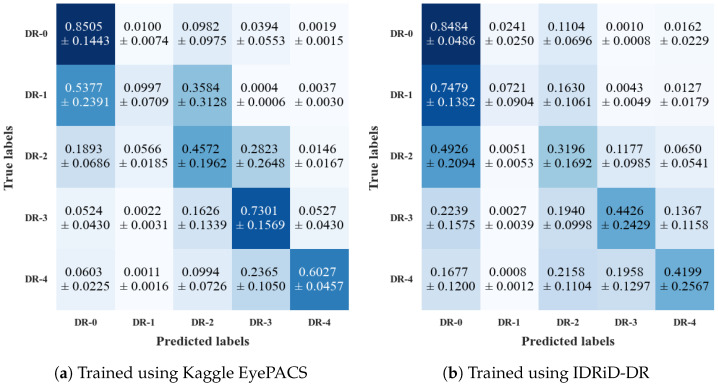
Confusion matrices of our ADRCs evaluated on the Kaggle EyePACS, IDRiD, and Messidor-2 datasets. Each subfigure shows mean ± std over each class of the three datasets.

The results also show that the ADRC trained on the Kaggle EyePACS dataset achieved the highest overall performance across all datasets. The reason may be attributed to the large size of the training set of the Kaggle EyePACS dataset (a total of 35,126 images). The ADRC achieved its highest performance across most evaluation metrics when it was evaluated on the test set of the Kaggle EyePACS dataset. On the other hand, the performance on the IDRiD-DR dataset was significantly low, with an accuracy of only 0.4951, although the drop in AUC was not substantial. The F1 score for this dataset was also the lowest, implying poor precision and recall. We also observed a moderate performance with an accuracy of 0.7466 and an AUC of 0.8674 on the Messidor-2 dataset. The specificity for this dataset was significantly higher than on the Kaggle EyePACS dataset. Figure 3a shows the confusion matrix of the model trained on the Kaggle EyePACS dataset, evaluated over the three mentioned datasets. It highlights the overall class-wise performance for each of the DR grades. We observed that the performance for DR-0, DR-3, and DR-4 was the highest, whereas that for DR-1 and DR-2 was the lowest. The model failed to correctly identify DR-1 in most instances. Most of the time, the model predicted them as DR-0 or DR-2. The detection performance for the DR-2 grade was moderate, with nearly half (45.72%) of the overall images correctly identified. Most of the misclassified DR-2 images were classified as DR-3, with a smaller number classified as DR-1.

The ADRC trained using the IDRiD-DR dataset achieved the highest score in its test set but had lower performance on the other datasets compared to the ADRC trained using the Kaggle EyePACS dataset. This is not unexpected, as the IDRiD-DR training set is much smaller than the Kaggle EyePACS training set, with only 413 heavily class-imbalanced images. Figure 3b shows the confusion matrix of the ADRC trained on the IDRiD-DR dataset. We found that the ADRC had a bias towards the DR-0 grade. Except for DR-0, the successful detection of no other grades exceeded 45%. The ADRC predicted most of the images from the DR-0 to DR-2 grades as DR-0. Over 20% of the DR-3 and DR-4 images were misclassified as DR-0 and DR-2, respectively.

The performance differences of our ADRCs across the DR grades may be attributed to class imbalance in the Kaggle EyePACS and IDRiD-DR datasets. Both datasets contain a large proportion of DR-0 images, with substantially fewer samples in the higher grades, which biased the ADRCs towards DR-0, particularly in the IDRiD-DR dataset. However, due to the comparatively larger number of non–DR-0 images in Kaggle EyePACS, the ADRC trained on this dataset was better able to learn discriminative features for the other DR grades than the ADRC trained on IDRiD-DR. For both of our ADRCs, we observed high standard deviations in the performance for certain DR grades. This suggests substantial variability in detection across datasets. The variability may be attributed to differences in image quality, grading distributions, and other dataset-specific characteristics.

### 6.2. Statistical Analysis of Prediction Scores

Table 6 contains detailed statistics of our ADRCs’ prediction scores. The table includes the number of predictions in each class, along with the minimum, maximum, mean, median, and standard deviation values for both the correct and incorrect predictions. Note that scores of correct predictions belong to the target class, whereas scores of incorrect predictions belong to a class other than the target class.

As shown in Table 6, for our five-class ADRCs, we observed that the model trained on the Kaggle EyePACS dataset had more correct predictions than incorrect ones for nearly all severity grades of the three datasets. For accurate predictions, the mean and median probability scores of DR-0 and DR-4 were the highest. For example, in the case of DR-0 of the Kaggle EyePACS dataset, the mean and median probability scores were 0.9422 and 0.9800 for correct predictions, and 0.6361 and 0.6107 for incorrect predictions, respectively. Similarly, for DR-4, the corresponding scores were 0.9118 and 0.9957 for correct predictions and 0.7212 and 0.7353 for incorrect predictions. This suggests that when the ADRC made correct predictions, it was more confident in its predictions than when it made incorrect ones. Conversely, DR-1 had the lowest mean and median probability scores for correct predictions. Moreover, it had the highest mean and median confidence for incorrect predictions. For example, the mean and median probability scores for correct predictions were 0.6800 and 0.6824, respectively, compared to 0.8523 and 0.9258 for incorrect predictions on the Kaggle EyePACS dataset. This indicates that the model was particularly confused when predicting DR-1. We also observed that the model was unable to correctly predict any of the DR-1 images in the IDRiD-DR dataset. The overall performance was also significantly lower for this dataset. Lastly, in the case of the Messidor-2 dataset, we observed comparable performance across all grades to that of the Kaggle EyePACS dataset. For our IDRiD-DR dataset-based ADRC, we observed decent performance on its own test set but much poorer performance on the other two datasets. The number of incorrect predictions for every DR grade was substantially higher. We again observed poor mean and median probability scores for the DR-1 grade for correct predictions and higher scores for incorrect predictions.

### 6.3. Performance of Segmentation Models

We trained six segmentation models using the datasets mentioned in Table 3. Since in many cases, multiple areas in the generated masks were detected as the optic disc and macula, which can only be one, we applied image processing techniques to retain only one optic disc and macula per image. We did not use any post-processing techniques while generating the four lesion masks. The performances of our six segmentation models are given in Table 7. We observed that segmenting the optic disc and macula was easier than segmenting four types of lesions. We also observed that the segmentation model failed to segment microaneurysms more often than other lesions.

## 7. ADRCs’ Decision Analysis Using XAI Techniques

### 7.1. Manual Analysis

**Figure 4:**
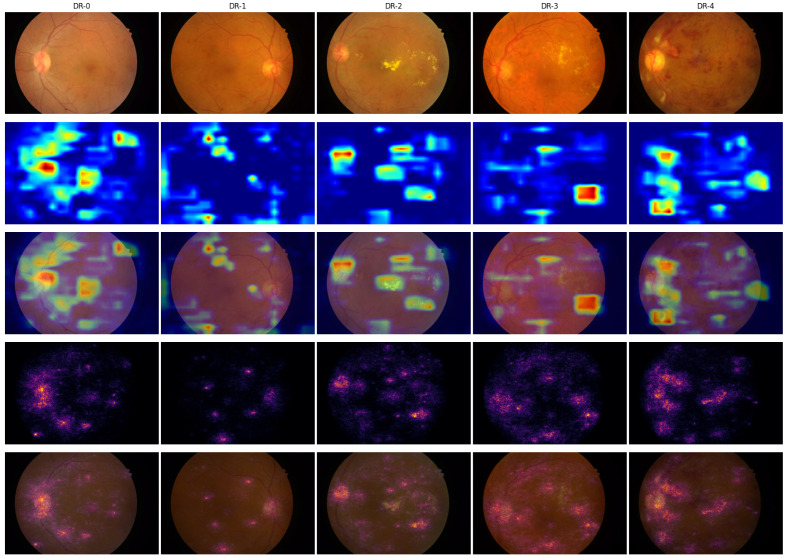
Visualization of Grad-CAM and IG for sample images from the IDRiD-DR dataset. Each row shows the original image, the Grad-CAM heatmap and overlay, and the IG heatmap and overlay. The five columns correspond to images from DR-0 through DR-4 grades, where the ADRC made correct predictions in all cases except DR-1.

Using the IDRiD-DR dataset, we evaluated manually whether our ADRCs activated around the correct regions in fundus images when predicting DR grades. We employed Grad-CAM and IG heatmaps from our five-class ADRC, trained on the Kaggle EyePACS dataset. We then quantified the number of images in each grade where the ADRC highlighted relevant regions, such as microaneurysms, exudates, hemorrhages, neovascularization, the optic disc, or the macula. If there were more than one such region, we counted whether at least one of them was highlighted. Figure 4 shows one image from each grade with corresponding Grad-CAM and IG heatmaps and overlays, where the ADRC predicted all grades correctly except DR-1. The results of our analysis on the entire dataset, along with the number of correct and incorrect predictions, are summarized in Table 8.

According to the ICDR grading scheme, DR-0 cases do not contain any abnormalities. For the DR-0 example image (column 1 of Figure 4), both Grad-CAM and IG heatmaps exhibited the strongest activation around the macula and optic disc regions. Additional activation was observed along the retinal blood vessels, while the IG heatmap also highlighted some unrelated regions. Across all the analyzed images, we consistently observed the same phenomena in the majority of cases in both the Grad-CAM and IG heatmaps (rows 1 and 6 in Table 8). In the ICDR grading scheme, the presence of only microaneurysms characterizes the DR-1 grade. For the example representing DR-1 (column 2), the Grad-CAM heatmap had the strongest activations appearing in two distinct regions containing microaneurysms: one at the top and another at the bottom of the retina. Although the image contained more than two microaneurysms, only these two were consistently highlighted. In the IG heatmap, further activation appeared in other areas, some of which corresponded to microaneurysms, while others did not. Upon analyzing all the images in a similar manner, we found that the optic disc and macula regions were most frequently highlighted in both the Grad-CAM and IG heatmaps. The ADRC also highlighted microaneurysms in four out of five images in the Grad-CAM heatmap and in three out of five images in the IG heatmap. Despite these relevant activations, the ADRC still failed to classify the images correctly. As presented in Table 6, the mean and median probability scores for this grade remained very low, even in cases of misclassification. This further underscores the difficulty the ADRC faced, despite focusing on clinically important lesions. According to the ICDR grading scheme, DR-2 is characterized by the presence of one or more microaneurysms, retinal dot or blot hemorrhages, and hard or soft exudates. In the DR-2 example (column 3), the Grad-CAM heatmap strongly activated around exudate regions and the optic disc. The IG heatmap highlighted the same regions while also highlighting additional areas. Some of these areas also contained additional lesions, such as exudates and microaneurysms. Analyzing all the images, we found that the ADRC was able to highlight regions of exudates in 21 out of 32 total images in the Grad-CAM and 23 out of 32 images in the IG heatmaps, respectively. However, it still lacked confidence (see Table 6) and misclassified the majority of the images. The ICDR grading scale defines DR-3 as a stage in which each of the four quadrants contains more than 20 intraretinal hemorrhages, with either definite venous beading in at least two quadrants or prominent intraretinal microvascular abnormalities in at least one quadrant, without signs of proliferative retinopathy. For the DR-3 example image (column 4), we found the highest activation on a hemorrhage for the Grad-CAM heatmaps. The IG heatmaps also highlighted the same hemorrhage in the highest activated regions, along with some exudates. Analyzing all the images, we again observed that the ADRCs had activation on exudates and hemorrhages in most of the instances (both for Grad-CAM and IG heatmaps). These lesions are relevant for the DR-3 grade. We also found some of the images to have activation on the optic disc, macula, and microaneurysms for the Grad-CAM and IG heatmaps. According to the ICDR scale, DR-4 is characterized by the presence of neovascularization or vitreous or preretinal hemorrhages. In the DR-4 example (column 5), the ADRC primarily focused on areas containing neovascularization in both Grad-CAM and IG heatmaps. The ADRC also showed activations in other regions containing retinal hemorrhages. From the analysis of all images, we observed that the ADRC activated around hemorrhages in 12 out of 13 instances in both the Grad-CAM and IG heatmaps. The ADRC also detected and highlighted neovascularization in eight images but correctly classified only half of them. In the IG heatmap specifically, activations were observed around the neovascularization region in two correctly predicted images and one misclassified image. In both heatmap types, the ADRC additionally highlighted other lesions such as microaneurysms and exudates, while the IG heatmap further showed activations around the optic disc and macula regions.

Although our manual observation revealed that the ADRC could identify relevant lesions in most images, it still failed to classify a significant number of DR-1 and DR-2 images accurately. The boundary between these grades and the adjacent grades is subtle and often challenging, even for HRPs. We also observed that although the ADRCs’ decision-making is influenced by multiple retinal regions, including areas with lesions and non-lesions, it did not consistently follow the ICDR grading criteria.

**Figure 5:**
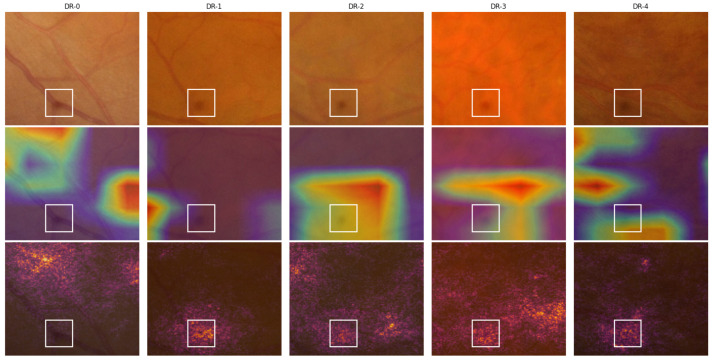
A close-up view of the lower region of the fundus images from Figure 4 showcasing a spot caused by an imaging equipment flaw. Here, row 1 contains the cropped position of the original images, and rows 2 and 3 contain the Grad-CAM and IG overlays, respectively. The white boxes highlight the identified spots.

In most of the IDRiD-DR test images, we further noted spots that were in the same regions of the images. The observed spots were not related to any retinal abnormalities and appeared to be caused by imperfections in the imaging equipment (e.g., dust or smudges on the fundus camera lens). For example, there is a spot that appears consistently in the bottom portion of all the example images of row 1 in Figure 4. A close-up view of this region with the heatmap overlays is shown in Figure 5. The spot is not clearly visible in the DR-1 image because it is on a blood vessel. The ADRC also activated around these imperfections, particularly in the IG heatmaps, for all DR grades except DR-1. Similar spots like this one may have contributed to the confusion of the ADRC and may have resulted in the poor overall performance.

### 7.2. Automatic Analysis

As described in Section 7.1, we conducted a manual analysis of the predictions of our ADRC model trained on the Kaggle EyePACS dataset. Our manual analysis involved careful examination of the areas highlighted by the Grad-CAM and Integrated Gradients (IGs) heatmaps for each fundus image in the IDRiD-DR test set. It required a significant amount of time and effort, and it is not feasible to analyze the performance of all our ADRCs, especially with large test sets. Therefore, we propose a new method for assessing the interpretability of ADRCs using the binary masks of different lesions (i.e., microaneurysms, soft exudates, hard exudates, and hemorrhages) and landmarks (i.e., optic disc and macula) automatically. We utilized two datasets for this analysis: the IDRiD-DR test set and the Retinal-Lesions dataset.

**Figure 6:**
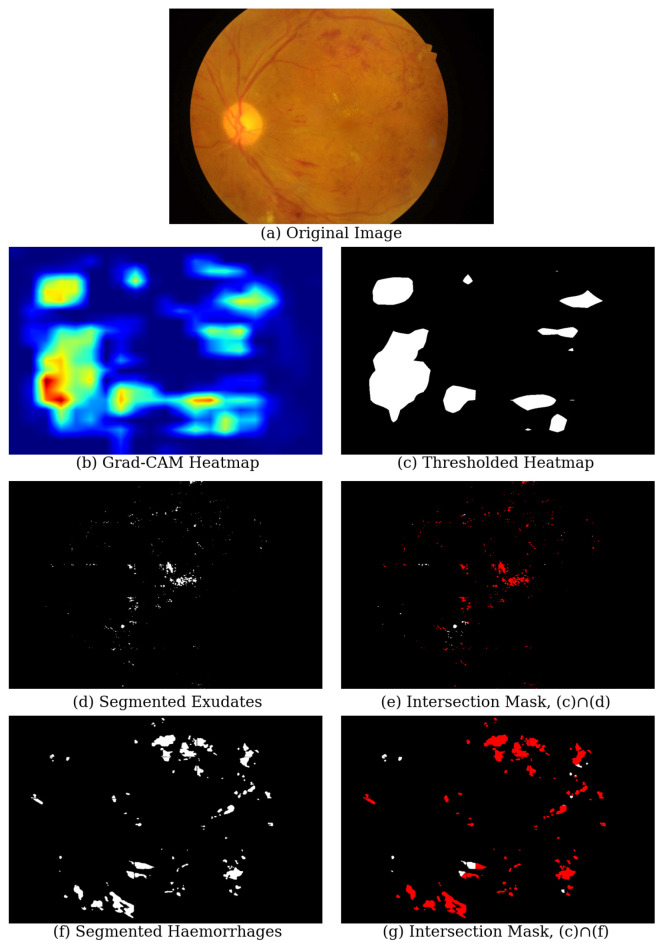
Our procedure of automatically evaluating ADRC interpretability. We demonstrate the process of interpreting the Grad-CAM activation around the exudates and hemorrhages. Here: (**a**) original fundus image, (**b**) Grad-CAM heatmap (with color-map) highlighting the ADRC-activated regions of the fundus image, (**c**) binary thresholded heatmap showing the highest activations (90th percentile), (**d**,**f**) segmented lesion masks from our trained segmentation models (exudates and hemorrhages), and (**e**,**g**) intersection map from the logical AND operation between the thresholded heatmap and lesion masks. The white regions represent overlapping pixels, whereas red regions correspond to non-overlapping regions. The non-overlapping regions refer to regions with lesion masks but no heatmap activation.

To automatically analyze the interpretability of our ADRCs, we checked how often the regions highlighted by Grad-CAM and IG correspond to clinically relevant lesions using the method described in Section 4. The overlapped heatmap and segmentation mask suggest that the ADRC focuses on that particular area while making predictions. It shows that the ADRC’s decisions are clinically meaningful, which increases trust among medical professionals. By comparing the high-activation regions with the output of our trained segmentation models, we were able to quantify whether the ADRCs consistently focus on meaningful pathological areas. Figure 6 illustrates our process for automatically assessing the interpretability of an ADRC prediction by comparing its Grad-CAM activations with segmented lesion masks. We observe that there are only a few overlapping regions. The IoU values for the overlapping areas in Figure 6e and Figure 6g are 0.0052 and 0.0188, respectively. This result was expected, since the applied XAI methods, Grad-CAM and IG, selectively emphasized the regions most influential for predicting the DR grade rather than capturing all pathological areas. Both methods highlighted regions that contributed most to the ADRCs’ decision, but those regions may not necessarily align perfectly with localized lesions (as shown in Figure 4). Grad-CAM produced coarse and low-resolution heatmaps. When the heatmap was resized to the original image’s size, the activation spread across broader areas. This made fine-grained overlap with small lesions unlikely. On the other hand, IG captured pixel-level sensitivity of the prediction score. However, the highlighted pixels often extended beyond the precise lesion boundaries rather than only the annotated lesion itself. Therefore, for our analysis, we only considered whether there was any overlap between the high-activation regions and the lesion masks, instead of the amount of overlap.

The results of our approach for automatic analysis are summarized in Table 9 and Table 10. Our approach may have two main limitations. The first limitation is that we only trained automatic segmentation models for four types of lesions. Segmentation models of other lesions or pathologies responsible for higher levels of DR, such as retinal hemorrhages, preretinal hemorrhage, vitreous hemorrhage, fibrous proliferation, and neovascularization, were not used due to the lack of exact pixel-wise annotated training data for such segmentation tasks. The second limitation is that the intersected areas created by the false positives in the segmentation masks may lead to the incorrect conclusion that the ADRC highlighted a lesion that did not exist. To overcome the limitations, we used the ground truth masks of the eight lesion types provided by the Retinal-Lesions dataset. The results are compiled in Table 11. We found that the results are similar to those of our automatic approach.

As shown in Table 9, we observed that exudates and hemorrhages influenced the decision-making process for the DR-0 images of the IDRiD-DR dataset. Similarly, for the Retinal-Lesion (A), we observed the impact of microaneurysms, exudates, and hemorrhages for classifying images belonging to DR-0 (see Table 11). However, according to the ICDR grading scale, images of DR-0 should not have any abnormalities; i.e., the presence of exudates and hemorrhages is not possible for DR-0. One reason for this phenomenon could be that our trained segmentation models were weak and incorrectly recognized normal areas as exudates and hemorrhages. Another possible reason is the incorrect ground truth labels in the Kaggle EyePACS dataset, which may have been taken from the Retinal-Lesion dataset. Besides these, we observed that both the optic disc and macula regions had heated areas in the Grad-CAM and IG heatmaps. In Table 10, we observed uniform lesion detection across the correct and incorrect Retinal-Lesions (B) set predictions. In Table 11, we found that the semi-automatic analysis based on manually segmented ground truth masks was closely aligned with the fully automatic analysis, which was based on masks generated using the trained segmentation models for microaneurysms and hemorrhages.

Table 12 presents Cohen’s kappa scores quantifying the agreement between our manual and automatic analysis on the IDRiD-DR test set. It was created using the common features from Table 8 and Table 9. The results revealed significant disagreement between our manual and automatic analyses, particularly in cases where predictions by the ADRC were incorrect. In our manual analysis, we carefully inspected the overlays to identify whether high-activation regions overlapped with the presence of lesions. In contrast, our automatic analysis relied on overlap calculations using the method described in Section 4. The limited robustness of the segmentation models, which often produced false detections (including in normal fundus images) and missed true lesions, largely accounts for the low agreement observed between the two approaches.

From Table 9, Table 10 and Table 11, we notice that our ADRCs recognized lesions, i.e., signs of diabetes, quite well. However, they did not completely rely on these lesions to determine the level of DR for fundus images. Besides different types of lesions, we found that other parts of the retina, such as the optic disc, macula, and sometimes blood vessels, also influenced ADRCs’ decisions. Moreover, we observed that ADRCs did not consider all relevant lesions when predicting the DR grade. We also observed a notable disagreement between our manual and automatic analyses (see Table 12). To increase agreement, more advanced segmentation models, trained on larger and more diverse datasets with improved lesion annotations, should be employed, which would enhance the reliability of the automatic interpretability analysis. We found that the ADRC’s decision-making procedure regarding the DR severity level is quite complicated. While our analysis has clarified some aspects, more details will have to be analyzed in future work. Our ADRCs also showed some transferability issues across datasets. These will also need to be analyzed in future work through domain adaptation, advanced data augmentation, or other strategies. With our current understanding of DNNs’ working procedure, we could not determine the reasons behind incorrect predictions, despite observing that diabetic signs influenced the final decision in the same way as they do in correct predictions. We propose that non-lesion areas that influence the ADRCs’ prediction can help HRPs.

## 8. Conclusions

In this paper, we investigated whether deep neural network (DNN)-based Automatic Diabetic Retinopathy Classifiers (ADRCs) follow the same criteria as human retina professionals (HRPs). In general, HRPs follow the criteria listed in the International Clinical Diabetic Retinopathy (ICDR)’s grading scheme. To this end, we trained DNN-based ADRCs for DR severity classification and six segmentation models and generated binary masks for the optic disc, macula, and four kinds of abnormalities, such as microaneurysms, hard exudates, soft exudates, and hemorrhages. Our DNN-based ADRCs were able to detect normal fundus images with high accuracy, and severe and proliferative DR with reasonable accuracy. However, the ADRCs failed to detect retinas affected by mild and moderate DR. Our segmentation models showed moderate performance.

Using Gradient-weighted Class Activation Map (Grad-CAM) and Integrated Gradients (IGs) eXplainable Artificial Intelligence (XAI) techniques, we generated heatmaps from our best-performing ADRC. Using the generated segmentation masks and heatmaps, we automatically analyzed the decision of our ADRC. Our manual observation showed that while the ADRC identified relevant lesions in most cases, it struggled to accurately classify many DR-1 and DR-2 images due to their subtle boundaries with adjacent grades. Moreover, its decision-making, though influenced by both lesion and non-lesion regions, did not consistently align with the ICDR grading criteria. Our automatic analysis also revealed that while ADRCs recognize retinopathy lesions, their decisions are also influenced by non-lesion retinal regions. In the future, we will use advanced DNN-based models for building ADRCs and segmentation models. 

## Figures and Tables

**Table 1:** Datasets employed at each stage of our study.

Task	Datasets
Building ADRCs	Kaggle EyePACS [71,72], IDRiD-DR [73], Messidor-2 [74]
Building Segmentation Models	IDRiD [73], E-ophtha [75], PALM [76]
Analyzing ADRCs’ Decision	IDRiD-DR [73], Retinal-Lesions [77]

**Table 2:** Distribution of images in the DR severity classification datasets used for building ADRCs.

Dataset Name	Train						Test						Total
	**DR-0**	**DR-1**	**DR-2**	**DR-3**	**DR-4**	**Subtotal**	**DR-0**	**DR-1**	**DR-2**	**DR-3**	**DR-4**	**Subtotal**	
Kaggle EyePACS	25,810	2443	5292	873	708	35,126	39,533	3762	7861	1214	1206	53,576	**88,702**
IDRiD-DR	134	20	136	74	49	413	34	5	32	19	13	103	**516**
Messidor-2	-	-	-	-	-	-	1017	270	347	75	35	1744	**1744**

**Table 3:** Distribution of images used for building segmentation models. Here, Type: type of segmentation area, #Patches: number of patches per image, #Train: number of training images, #Valid: number of validation images, #Test: number of test images, MA: microaneurysms, EX: hard exudates, HE: haemorrhage, SE: soft exudates, OD: optic disc, MC: macula.

Type	Datasets	#Patches	#Train	#Valid	#Test
MA	IDRiD + E-ophtha	25	155	35	39
EX	IDRiD + E-ophtha	25	86	20	22
HE	IDRiD	25	53	13	14
SE	IDRiD	25	26	7	7
OD	IDRiD + PALM	1	289	64	73
MC	PALM	1	234	52	59

**Table 4:** Distribution of images in our datasets used for ADRCs’ decision analysis.

Dataset	DR-0	DR-1	DR-2	DR-3	DR-4	Total
IDRiD-DR	34	5	32	19	13	103
Retinal-Lesions (A)	67	184	561	120	45	977
Retinal-Lesions (B)	-	-	415	136	65	616

**Table 5:** Performance of our DNN-based ADRCs for DR severity classification.

Train Dataset	Test Dataset	Accuracy	F1 Score	Specificity	AUC
Kaggle EyePACS	Kaggle EyePACS	0.8287	0.8102	0.7246	0.8785
	IDRiD-DR	0.4951	0.4765	0.8414	0.8352
	Messidor-2	0.7466	0.7120	0.7844	0.8674
IDRiD-DR	Kaggle EyePACS	0.7042	0.6507	0.4492	0.6589
	IDRiD-DR	0.6214	0.6089	0.8655	0.8412
	Messidor-2	0.5826	0.5338	0.6575	0.7020

**Table 6:** Statistical analysis of the probability scores for correct and incorrect predictions of our DNN-based ADRCs for DR severity classification.

TrainDB	TestDB	Grade	Correct Prediction						Incorrect Prediction					
			**Count**	**Min**	**Max**	**Mean**	**Median**	**Std**	**Count**	**Min**	**Max**	**Mean**	**Median**	**Std**
Kaggle EyePACS	Kaggle EyePACS	0	38,199	0.2978	1.0000	0.9422	0.9800	0.0946	1334	0.2889	1.0000	0.6361	0.6107	0.1624
		1	596	0.3153	0.9840	0.6800	0.6824	0.1526	3166	0.2838	1.0000	0.8523	0.9258	0.1613
		2	4211	0.2988	0.9988	0.7832	0.8195	0.1606	3650	0.2977	1.0000	0.7616	0.7893	0.1841
		3	617	0.3458	0.9986	0.7663	0.7892	0.1599	597	0.3392	1.0000	0.7304	0.7376	0.1685
		4	773	0.3598	1.0000	0.9118	0.9957	0.1476	433	0.3192	0.9990	0.7212	0.7353	0.1738
	IDRiD-DR	0	22	0.5599	0.9990	0.9024	0.9313	0.1270	12	0.3918	0.6568	0.4961	0.4773	0.0895
		1	0	-	-	-	-	-	5	0.3893	0.9932	0.6285	0.5188	0.2603
		2	6	0.5877	0.8786	0.6890	0.6447	0.1233	26	0.4320	0.9823	0.7082	0.7212	0.1448
		3	16	0.4993	0.9658	0.7695	0.8338	0.1617	3	0.5036	0.9936	0.7712	0.8163	0.2481
		4	7	0.7129	0.9714	0.8496	0.8585	0.0810	6	0.5334	0.8767	0.6868	0.6334	0.1484
	Messidor-2	0	954	0.3746	0.9997	0.9283	0.9749	0.1112	63	0.3703	0.9613	0.6420	0.6235	0.1516
		1	38	0.3634	0.7954	0.5915	0.6150	0.1207	232	0.3661	0.9992	0.8076	0.8644	0.1775
		2	225	0.3180	0.9857	0.7269	0.7619	0.1581	122	0.3216	0.9979	0.7043	0.6930	0.1786
		3	63	0.5104	0.9971	0.8378	0.8931	0.1475	12	0.4974	0.9490	0.6705	0.7025	0.1558
		4	22	0.4267	0.9999	0.8174	0.9691	0.2231	13	0.4046	0.9738	0.6919	0.6238	0.2094
IDRiD-DR	Kaggle EyePACS	0	36,222	0.2848	1.0000	0.9687	0.9988	0.0853	3311	0.2825	1.0000	0.7555	0.7810	0.1939
		1	34	0.3771	0.9556	0.6723	0.6863	0.1775	3728	0.2962	1.0000	0.9564	0.9984	0.1070
		2	631	0.3211	0.9993	0.7145	0.7135	0.1764	7230	0.2881	1.0000	0.9101	0.9891	0.1496
		3	134	0.3480	0.9971	0.7244	0.7242	0.1766	1080	0.3007	1.0000	0.8238	0.8938	0.1846
		4	708	0.2761	1.0000	0.8959	0.9723	0.1474	498	0.2869	1.0000	0.8021	0.8690	0.1927
	IDRiD-DR	0	28	0.4664	1.0000	0.9627	0.9962	0.1011	6	0.5833	0.9436	0.7346	0.6735	0.1662
		1	1	0.6733	0.6733	0.6733	0.6733	0.0000	4	0.7935	0.9984	0.9250	0.9540	0.0948
		2	14	0.4390	1.0000	0.8432	0.9330	0.1813	18	0.5406	0.9997	0.8794	0.9420	0.1423
		3	13	0.5118	0.9866	0.8424	0.8855	0.1332	6	0.5114	0.9965	0.7682	0.8096	0.2134
		4	8	0.6453	0.9993	0.8231	0.8198	0.1417	5	0.4572	0.8498	0.6560	0.6431	0.1709
	Messidor-2	0	819	0.3463	1.0000	0.9308	0.9931	0.1291	198	0.3577	0.9995	0.8039	0.8493	0.1718
		1	2	0.3396	0.4064	0.3730	0.3730	0.0472	268	0.2867	1.0000	0.8680	0.9346	0.1602
		2	153	0.3798	0.9992	0.8226	0.8694	0.1586	194	0.3337	0.9999	0.8437	0.9468	0.1847
		3	40	0.4838	0.9991	0.8317	0.9486	0.1870	35	0.4035	0.9994	0.7386	0.7425	0.1873
		4	2	0.4735	0.7559	0.6147	0.6147	0.1997	33	0.4775	0.9967	0.7858	0.8327	0.1733

**Table 7:** Mean Intersection-over-Union (mIoU) scores of our segmentation models. In Round 1, the models were trained on the training set and evaluated on the test set. In Round 2, the models were retrained on the combined training and test sets. In both rounds, the same validation set was used. Here, MA: microaneurysms, EX: hard exudates, HE: hemorrhage, SE: soft exudates, OD: optic disc, MC: macula.

Segmentation Category	Round 1		Round 2
	**Val. Set**	**Test Set**	**Val. Set**
MA	0.6042	0.5800	0.5996
EX	0.7904	0.7608	0.8240
HE	0.7169	0.6942	0.7344
SE	0.7513	0.6411	0.7659
OD	0.9162	0.9229	0.9382
MC	0.8159	0.8237	0.8229

**Table 8:** Manual analysis of the highlighted regions in Grad-CAM and IG heatmaps for our ADRC trained using the Kaggle EyePACS dataset. The analysis was performed on the IDRiD-DR test set, showing the number of images for which the ADRC paid attention to various DR lesions across the dataset. Here, # Images: total images per DR grade, OD: optic disc, MC: macula, HE: hemorrhage, EX: exudate, MA: microaneurysms, NV: neovascularization, OT: other areas of the fundus image.

Method	DR	#	Correct Prediction								Incorrect Prediction							
	**Grade**	**Images**	**Total**	**MA**	**EX**	**HE**	**NV**	**MC**	**OD**	**OT**	**Total**	**MA**	**EX**	**HE**	**NV**	**MC**	**OD**	**OT**
Grad-CAM	0	34	22	0	0	0	0	19	11	22	12	0	0	0	0	8	2	12
	1	5	0	-	-	-	-	-	-	-	5	4	0	0	0	0	0	1
	2	32	6	5	3	0	0	0	0	1	26	18	18	9	0	0	1	10
	3	19	16	16	14	10	0	1	6	11	3	2	1	1	0	0	0	1
	4	13	7	5	6	6	4	0	0	1	6	3	3	6	4	0	1	3
IG	0	34	22	0	0	0	0	9	13	20	12	0	0	0	0	7	1	12
	1	5	0	-	-	-	-	-	-	-	5	3	0	0	0	2	1	5
	2	32	6	5	4	0	0	5	4	6	26	25	19	9	0	13	22	24
	3	19	16	16	14	12	0	9	16	16	3	3	1	1	0	2	2	3
	4	13	7	6	5	7	2	5	7	6	6	6	4	5	1	4	6	6

**Table 9:** Automatic analysis of the ADRC highlighted areas in Grad-CAM and IG heatmaps of the IDRiD-DR test set using automatic segmentation models. It displays the number of IDRiD-DR images where the ADRC focused on various DR lesions and landmarks, as identified by masks generated using six trained segmentation models. Here, # Images: total images per DR grade, MA: microaneurysm, SE: soft exudate, EX: hard exudate, HE: hemorrhage, MC: macula, and OD: optic disc.

Method	Class ID	# Images	Correct Prediction							Incorrect Prediction						
			**Total**	**MA**	**SE**	**EX**	**HE**	**MC**	**OD**	**Total**	**MA**	**SE**	**EX**	**HE**	**MC**	**OD**
Grad-CAM	0	34	22	8	11	20	16	21	16	12	7	2	9	7	11	5
	1	5	0	-	-	-	-	-	-	5	3	0	4	4	2	5
	2	32	6	4	3	6	6	5	6	26	18	14	25	21	14	26
	3	19	16	13	8	16	16	2	15	3	2	1	2	1	2	3
	4	13	7	5	5	6	7	3	7	6	5	4	6	6	1	4
IG	0	34	22	14	12	17	18	22	22	12	11	6	8	11	11	12
	1	5	0	-	-	-	-	-	-	5	4	0	4	5	5	5
	2	32	6	5	2	6	5	6	6	26	23	17	24	25	22	26
	3	19	16	16	12	16	16	12	16	3	3	0	2	2	2	3
	4	13	7	7	5	6	7	7	7	6	5	6	6	6	3	6

**Table 10:** Automatic analysis of the highlighted areas in Grad-CAM and IG heatmaps of the Retinal-Lesions (B) dataset. It shows the number of Retinal-Lesions (B) images where the ADRC focused on various DR lesions and landmarks located by masks generated using six trained segmentation models. Here, # Images: total images per DR grade, MA: microaneurysm, SE: soft exudate, EX: hard exudate, HE: hemorrhage, MC: macula, and OD: optic disc.

Method	Class ID	# Images	Correct Prediction							Incorrect Prediction						
			**Total**	**MA**	**SE**	**EX**	**HE**	**MC**	**OD**	**Total**	**MA**	**SE**	**EX**	**HE**	**MC**	**OD**
Grad-CAM	2	415	238	190	157	206	189	111	229	177	132	117	147	117	83	168
	3	136	74	72	56	71	71	16	30	62	53	44	56	53	21	38
	4	65	21	17	17	18	20	10	14	44	41	36	42	41	23	20
IG	2	415	238	224	200	228	216	230	234	177	144	135	156	137	168	177
	3	136	74	74	64	72	74	69	73	62	59	50	59	58	60	62
	4	65	21	19	18	20	21	16	21	44	41	36	44	42	40	44

**Table 11:** Semi-automatic analysis of the highlighted areas in Grad-CAM and IG heatmaps of the Retinal-Lesions dataset. It shows the number of images of the Retinal-Lesions dataset for which our ADRC (trained on the Kaggle EyePACS dataset) paid attention to various DR lesions and landmarks located by manually prepared ground truth binary masks. Here, # Images: total images per DR grade, MA: microaneurysm, SE: soft exudates, EX: hard exudate, rHE: retinal hemorrhage, pHE: preretinal hemorrhage, vHE: vitreous hemorrhage, FP: fibrous proliferation, NV: neovascularization, Subset: subset of Retinal-Lesion dataset.

Subset	Method	DR	#	Correct Prediction									Incorrect Prediction								
		**Grade**	**Images**	**Total**	**MA**	**SE**	**EX**	**rHE**	**vHE**	**pHE**	**FP**	**NV**	**Total**	**MA**	**SE**	**EX**	**rHE**	**vHE**	**pHE**	**FP**	**NV**
(A)	Grad-CAM	0	67	57	9	0	4	3	0	0	1	0	10	3	0	1	1	0	0	0	0
		1	184	33	26	0	0	1	0	0	0	0	151	75	2	6	13	0	0	0	0
		2	561	320	254	80	165	235	0	0	0	2	241	140	46	65	112	0	0	2	0
		3	120	94	80	44	70	92	0	0	0	0	26	23	8	15	20	0	0	0	0
		4	45	29	18	6	15	24	8	6	10	16	16	13	5	10	15	0	0	0	2
	IG	0	67	57	24	3	9	5	0	0	1	0	10	8	2	3	3	0	0	0	0
		1	184	33	32	0	3	4	0	0	0	0	151	106	6	28	17	0	0	0	0
		2	516	320	288	91	197	251	0	0	0	2	241	166	52	79	118	0	0	2	0
		3	120	94	87	52	76	92	0	0	0	0	26	24	10	16	22	0	0	0	0
		4	45	29	23	8	20	26	8	6	12	16	16	16	6	11	15	0	0	0	2
(B)	Grad-CAM	2	415	238	194	64	127	201	0	0	0	0	177	106	40	46	97	0	0	0	1
		3	136	74	57	45	55	72	0	0	0	0	62	53	24	29	53	0	0	0	1
		4	65	21	12	5	8	16	2	4	9	13	44	31	14	32	39	2	1	2	11
	IG	2	415	238	222	81	150	215	0	0	1	0	177	131	50	68	100	0	0	1	1
		3	136	74	69	48	59	73	0	0	0	1	62	59	26	33	54	0	0	0	1
		4	65	21	15	6	9	18	2	4	8	14	44	36	21	33	41	2	1	2	13

**Table 12:** Kappa scores for lesion detection agreement between our manual and automatic analyses of the highlighted areas in Grad-CAM and IG heatmaps across DR grades of the IDRiD-DR test set. Here, positive values indicate better-than-chance agreement, negative values indicate disagreement, zeros represent no agreement beyond chance, dashes (“– ”) denote missing cases, and “N/A” indicates that the kappa score is undefined due to no variability in the labels for that category.

Method	DR Grade	Correct Prediction					Incorrect Prediction				
		**MA**	**EX**	**HE**	**OD**	**MC**	**MA**	**EX**	**HE**	**OD**	**MC**
Grad-CAM	0	0.0000	0.0000	0.0000	0.3636	0.4634	0.0000	0.0000	0.0000	0.0625	0.3077
	1	–	–	–	–	–	−0.3636	0.0000	0.0000	0.0000	0.0000
	2	0.5714	0.0000	0.0000	0.0000	0.0000	−0.2639	0.0000	0.2239	0.0000	0.0000
	3	0.0000	0.0000	0.0000	0.0769	−0.0909	−0.5000	0.0000	1.000	0.0000	0.0000
	4	0.3000	0.0000	0.0000	0.0000	0.0000	−0.3333	0.0000	“N/A”	0.1818	0.0000
IG	0	0.0000	0.0000	0.0000	0.0000	0.0000	0.0000	0.0000	0.0000	0.0000	0.258
	1	–	–	–	–	–	0.5455	0.0000	0.0000	0.0000	0.0000
	2	1.0000	0.0000	0.0000	0.0000	0.0000	0.4694	0.1959	0.0415	0.0000	0.0000
	3	“N/A”	0.0000	0.0000	“N/A”	0.3333	“N/A”	0.4000	0.4000	0.0000	−0.5000
	4	0.0000	0.0000	“N/A”	“N/A”	0.0000	0.0000	0.0000	0.0000	“N/A”	0.6667

## Data Availability

The source code used in this research is publicly available at https://github.com/ahanaf019/Interpreting-Deep-Neural-Networks-in-Diabetic-Retinopathy-Grading, (accessed on 6 September 2025).
